# Recently discovered heteromeric enzymes in natural product biosynthesis

**DOI:** 10.1016/j.jbc.2025.108516

**Published:** 2025-04-17

**Authors:** Zhongtian Yu, Ikuro Abe

**Affiliations:** 1 Graduate School of Pharmaceutical Sciences, The University of Tokyo, Tokyo, Japan; 2 Collaborative Research Institute for Innovative Microbiology, The University of Tokyo, Tokyo, Japan

**Keywords:** biosynthesis, enzymes, protein-protein interaction, natural products, secondary metabolism

## Abstract

The abundant diversity and elegant complexity in the chemical structures of natural products have attracted vigorous investigations of the chemistry and enzymology underlying their biosynthetic processes over the past few decades. Among the biochemical events, the formation of complexes of heteromeric enzymes has been observed in the biosynthesis of several natural products and metabolic pathways. In this review, we aim to consolidate the recently discovered cases of heteromeric enzymes in natural product biosynthesis and metabolism, in order to clarify the genetic and structural bases leading to the formation of these heteromeric complexes and provide insights for the rational redesign of proteins in biosynthetic machineries.

Natural products, with their structural diversity and complexity, have played crucial roles in advancing the fields of medicinal chemistry and chemical biology (1, 2, 3, 4). Over the past few decades, researchers have focused extensively on understanding the biochemical and enzymatic mechanisms involved in the biosynthesis of natural products and metabolic pathways. In certain cases, enzyme complexes are employed to precisely coordinate catalysis. Advances in structural biology and physical biochemistry have greatly expanded the scope of understanding of biosynthetic protein-protein interactions in heteromeric enzymes.

Heteromeric enzymes are multiprotein assemblies consisting of nonidentical subunits. These complexes play essential roles in the biosynthetic pathways of various natural products. The formation of heteromeric complexes influences the stability, specificity, and catalytic efficiency of the enzymes involved (5, 6). As an example of a well-investigated biosynthetic enzyme cascade, the protein-protein interactions in polyketide synthase (PKS)-nonribosomal peptide synthetase hybrid assembly lines (7), especially the catalytic domains and carrier proteins (8), *trans*-AT PKSs (9), *cis*-AT PKSs (10) and type I/II PKSs, and fatty acid synthases (11, 12), have been broadly studied and discussed. Another interesting case is the formation of substrate channels (13, 14) by some heteromeric enzymes, such as the tryptophan synthase TrpAB (15) and the imidazole glycerol phosphate synthase HisHF (16, 17, 18). However, these enzymes are mostly involved in primary metabolism. In this review, we aim to summarize several representative cases of heteromeric enzymes that catalyze biosynthetic reactions of secondary metabolites, based on research over the past 7 years.

## Nonheme oxygenase TlxIJ in talaromyolides biosynthesis

**Figure 1:**
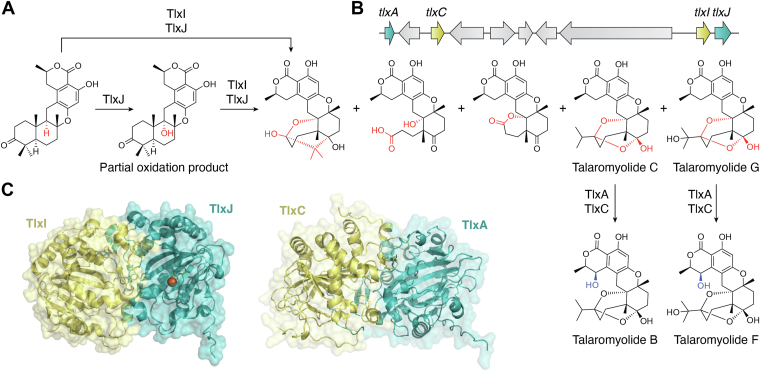
**Heterodimeric nonheme oxygenase TlxIJ.***A*, reaction catalyzed by TlxI and TlxJ. *B*, biosynthetic gene cluster for talaromyolides. *C*, complex structures of the cocrystal of TlxIJ with Fe/N-oxalylglycine (PDB ID: 7VBQ) and TlxAC (predicted by Alphafold 3). PDB, Protein Data Bank.

Talaromyolides, which are hexacyclic meroterpenoids, were isolated from the marine fungus *Talaromyces purpureogenus*. In 2021, the biosynthetic pathway of talaromyolides was characterized by heterologous expression and gene knockout studies by the Abe group (19). The talaromyolide biosynthetic gene cluster comprises ten genes, with four annotated as nonheme iron oxygenases (Fig. 1*B*). These enzymes play their roles in the later stages of biosynthesis, constructing the ketal scaffold and hydroxylating the C-4 carbon, followed by acetylation by TlxB to produce talaromyolides H and E. KO mutants of *tlxA* and *tlxC* resulted in the accumulation of the same biosynthetic intermediates, which was similar to the case of the *tlxI* and *tlxJ* KO mutants. *In vitro* experiments and sequence analyses revealed that TlxI lacks the arginine residue required for binding α-KG, and the loop A regions of TlxI and TlxC, which serve as caps for encapsulating substrates within the active sites, are shorter (20, 21, 22), leading to the hypothesis that TlxI and TlxC are dysfunctional as oxidases and instead serve as auxiliary proteins for other oxidases in the reactions. As expected, coincubations of TlxA/TlxC and TlxI/TlxJ with their substrates yielded their respective products. Pull-down experiments, electrophoretic mobility shift assays, and size-exclusion chromatography provided sufficient evidence for the heterodimerization between the TlxA/TlxC and TlxI/TlxJ enzymes. The crystal structure of TlxI/TlxJ complexed with Fe/N-oxalylglycine was solved (PDB ID: 7VBQ, Fig. 1*C*), revealing an extensive interface with buried surface area of 1950 Å^2^, as shown in Figure 1*C*. In fact, the overall structure of the heterodimer resembles the solution structure of AndA (22), a homodimeric αKG-dependent enzyme catalyzing an isomerization reaction in anditomin biosynthesis. In the TlxIJ heterodimer, the RMSD values for the Cα atoms and the amino acid sequence identities for TlxI are 1.8 Å and 33%, and for TlxJ, 1.4 Å and 40%, respectively. In AndA, four pairs of residues (L236 and T237′, P238 and N275′, D82 and Q278′, and D270 and Y139′) interact with each other by hydrogen bonding to support homodimer formation. However, only two pairs of these residues (L236 and T237′, P238 and N275′) are present at the same positions in the TlxIJ heterodimer. The other two pairs are not conserved, which prohibits TlxJ from forming a homodimer. The complex structure of heterodimeric TlxA/TlxC was predicted by Alphafold 3 (23) (Fig. 1*C*), and is similar to that of the TlxI/TlxJ heterodimer. It is noteworthy that TlxJ alone can also perform its catalytic role, yet the kinetic efficiency (*k*_*cat*_*/K*_*m*_ = 0.02 min^−1^ μM^−1^) was quite weak as compared to that of the TlxI/TlxJ heterodimer (*k*_*cat*_*/K*_*m*_ = 1.63 min^−1^ μM^−1^) and only a partial oxidation product could be observed, as shown in Figure 1*A*, because the loop B′ of TlxI is essential for substrate binding, even though it lacks the arginine residue for α-KG binding and is catalytically incompetent. These two pairs of heterodimeric nonheme iron enzymes in talaromyolide biosynthesis are absolutely unprecedented and provide novel insights for biosynthetic research.

Interestingly, a *tal* cluster recently identified in *Talaromyces pinophilus* LD-7 (24) is highly homologous to the *tlx* cluster, except that TlxI and TlxJ are replaced by their natural chimeric enzyme, TalA. The N-terminal domain of TalA is homologous to TlxI and the C-terminal domain resembles TlxJ. The RMSD values for the Cα atoms (TalA structure predicted by Alphafold 3) and the amino acid sequence identities of TalA and TlxI/TlxJ are 0.5 Å/0.6 Å and 72%/79%. The example of the discovery of TalA will elicit insightful considerations of the evolutionary process of natural fusion protein formation.

## Multinuclear nonheme iron dependent oxidative enzymes

**Figure 2:**
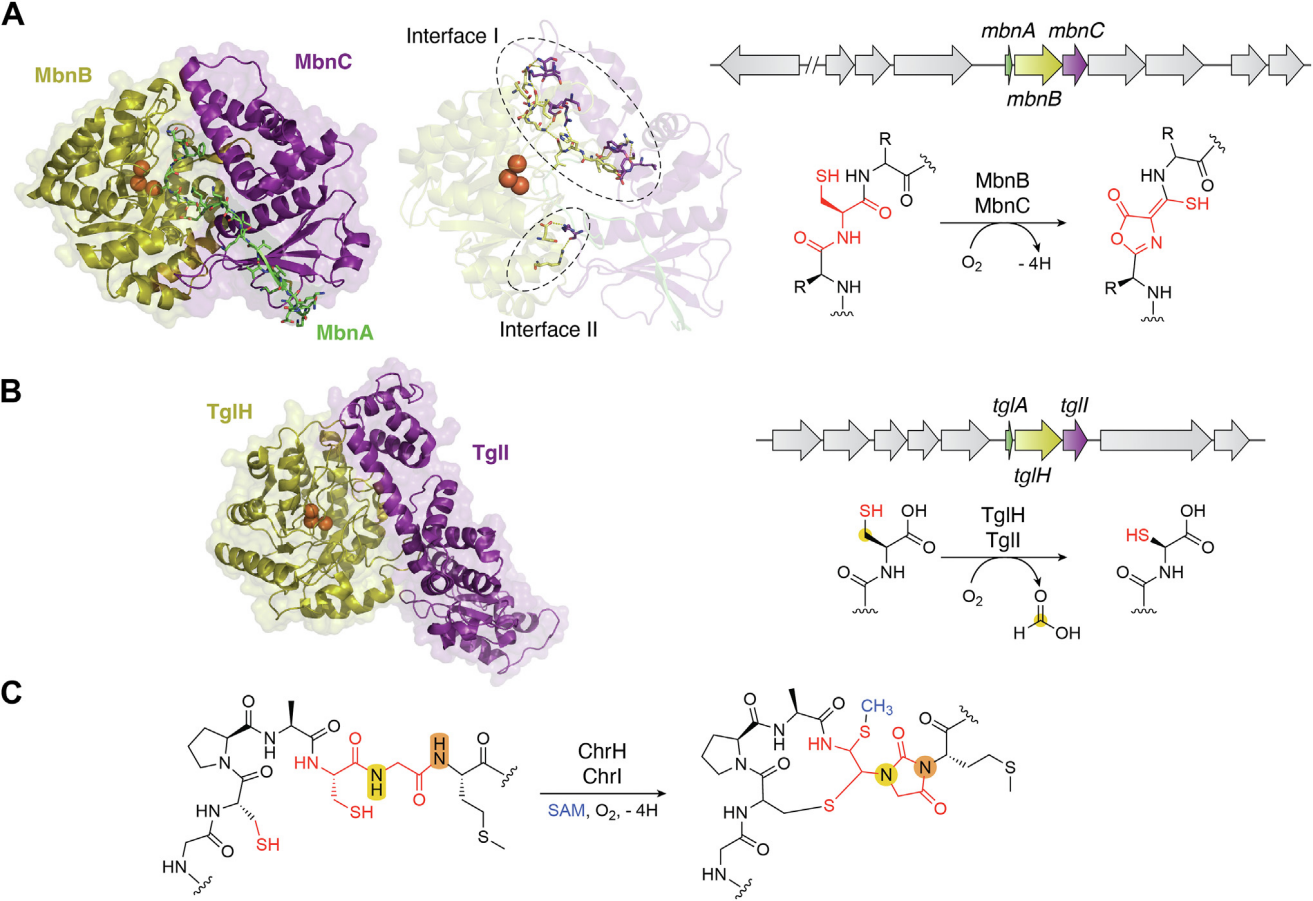
**The multinuclear nonheme iron-dependent oxidative enzymes.***A*, cocrystal structure of MbnBC with its peptide substrate MbnA (PDB ID: 7FC0), interaction interfaces I and II between MbnB and MbnC, the *mbn* cluster, and the oxidation reaction catalyzed by MbnBC. *B*, cocrystal structure of TglHI (PDB ID: 8HI8, buried surface area of dimerization interface is 1757 Å^2^), the *tgl* cluster, and the oxidation reaction catalyzed by TglHI. *C*, reaction catalyzed by the ChrHI heterodimer. PDB, Protein Data Bank.

As the very first identified Cu^2+^-binding natural products, the biosynthesis of methanobactins has been investigated in great detail. Methanobactins are ribosomally synthesized, posttranslationally modified peptides produced by methanotrophic bacteria, with therapeutic potential for Wilson's disease (25, 26). In 2018, the Rosenzweig group first reported the biosynthetic pathway of methanobactins (27). The translated product of the *mbnA* gene includes an N-terminal leader peptide to be cleaved later, as well as a C-terminal core peptide that directs posttranslational modifications. Once translated, the precursor peptide MbnA is oxidized at its conserved cysteine residues to generate an oxazolone ring and an adjacent thioamide group by the heterodimeric enzyme MbnBC. While trials of MbnB or MbnC expression both resulted in insoluble proteins, MbnBC was successfully obtained as soluble protein complex when coexpressed by a single vector in *Escherichia coli*. MbnB was the first biochemically identified member of the DUF692 protein family, and MbnC belongs to an uncharacterized protein family. A Mössbauer spectroscopic analysis revealed that MbnBC contains a mixture of triferric and diferric clusters (27). In the solved cocrystal structure of heterotrimeric MbnABC (PDB ID: 7FC0, Fig. 2*A*), the N-terminal domain of MbnC stretches into the middle of the extended loops of MbnB to form the interaction interface I. In addition, a hairpin loop and the linker loop between the N termini and C termini of MbnB reach out to MbnC to form the interaction interface II, with buried surface area of 1772 Å^2^ in total. The importance of the residues on these identified interfaces in the solved cocrystal structure was further verified by site-directed mutagenesis experiments, as well as a multiple sequence alignment analysis. The binding of MbnA to MbnBC is mainly supported by the MbnC subunit. The N terminus of MbnA forms three hydrogen bonds with MbnC as an antiparallel β-sheet, which enables MbnA to extend its core peptide part into the catalytic center of MbnB and the cysteine residue to be oxidized to coordinate with the iron ion. Binding affinity constants measured by isothermal titration calorimetry experiments indicated that the binding between MbnA and the MbnBC complex is relatively tight (*K*_d_ = 2.7 μM for RrMbnA/RrMbnBC and 3.0 μM for VcMbnA and VcMbnBC). The replacement of the reactive cysteine by alanine slightly decreased the binding affinity to a *K*_d_ of 4.3 μM. The truncation of the C-terminal core peptide weakened the interaction between MbnA and MbnBC to a *K*_d_ of 63.8 μM, while that of the N-terminal leader peptide almost completely abolished the interaction. These experimental results indicated that, in the biosynthesis of methanobactins, the formation of the MbnBC heterodimer facilitates the binding of its target substrate MbnA, with MbnB playing catalytic role and MbnC acting as a introducer to guide MbnA to correctly expose its cysteines within the active center of MbnB.

After the identification of the MbnBC complex, the second investigation of a DUF692 family protein was published in 2019 by the van der Donk group (28). In the biosynthetic gene cluster of 3-thioglutamate, the DUF692 family protein TglI is co-located with a homolog to the leader peptide-binding domains in RiPP biosynthetic enzymes, TglH. A cysteine was added to the C terminus of the precursor peptide TglA by the peptide aminoacyl-tRNA ligase (PEARL) family enzyme TglB, and then catalysis by the TglHI complex generated a β-carbon excised product, which was further modified by the carboxyl-*S*-adenosylmethionine synthase TglE and the carboxyl-*S*-adenosylmethionine-dependent methyltransferase TglF, and released by the membrane-bound protease TglG to generate 3-thioglutamate, which may act as a phytotoxin by interfering with glutamate receptor signaling (29). Like MbnB, TglH utilizes a tri-iron cluster in its active site to perform its catalytic activity. The solved crystal structure of heterodimeric TglHI (PDB ID: 8HI8, Fig. 2*B*) (30) revealed a similar arrangement to the MbnBC heterodimer, in which TglH undertakes a catalytic role and TglI acts as the TglA recognition subunit. Although they catalyze completely different reactions targeting a cysteine residue in each substrate peptide, the components of the TglHI and MbnBC heterodimers share similar biochemical functions. In 2023, a third heterocomplex of a DUF692 family protein and another partner protein for substrate peptide binding, ChrH and ChrI in the biosynthetic gene cluster of chryseobasin, was reported by the van der Donk group (31). ChrI assists ChrH in oxidizing its substrate to generate a macrocycle, an imidazolidinedione heterocycle, two thioaminals, and a thiomethyl group (Fig. 2*C*). Since MbnB, TglH, and ChrH all catalyze four-electron oxidative rearrangements toward cysteine residues of precursor peptide substrates without reducing agents, using a mixed-valent state iron ion cluster, the DUF692 family members were designated as multinuclear nonheme iron dependent oxidative enzymes (MNIOs). Although only a few other MNIO family enzymes have been investigated to date, it is quite likely that more MNIO family enzymes that form heterodimers with helper proteins will be found.

## Noncanonical KAS-III family lipstatin synthase LstAB

**Figure 3:**
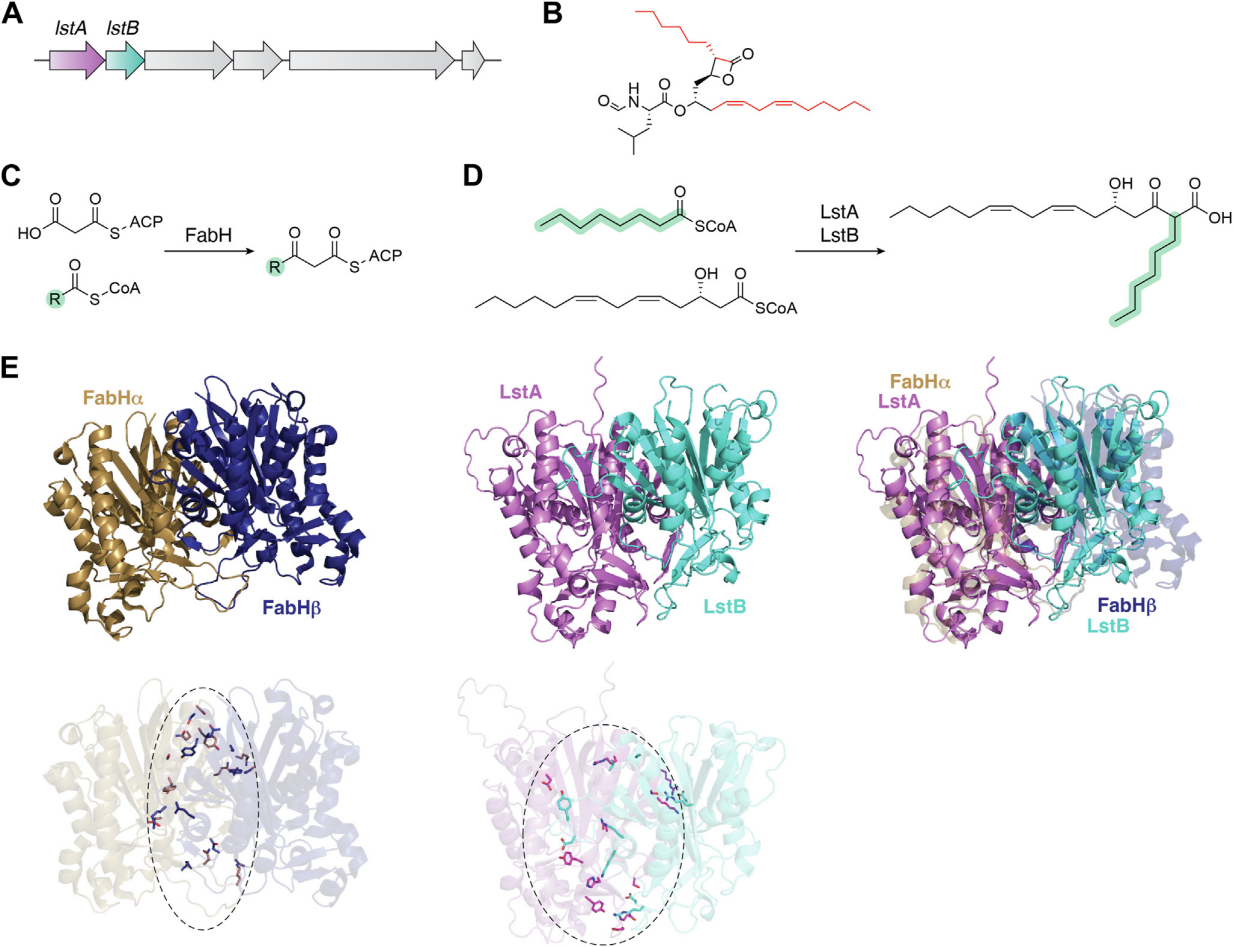
**Heterodimeric lipstatin synthase LstAB.***A*, biosynthetic gene cluster encoding lipstatin. *B*, chemical structure of lipstatin. Carbons from substrates of LstAB are highlighted in *red* to indicate the condensation reaction. *C*, condensation reactions catalyzed by FabH. *D*, condensation reaction catalyzed by LstAB. *E*, crystal structure of homodimeric β-ketoacyl-acyl carrier protein synthase-III FabH (PDB ID: 5BNS), heterodimeric complex model of LstA and LstB predicted by Alphafold 3, superimposition of these two structures and dimerization interfaces observed in the FabH crystal and the predicted model of LstAB. PDB, Protein Data Bank.

Another interesting case of a heterodimeric enzyme is LstAB in the biosynthesis of lipstatin, a natural lipase inhibitor produced by *Streptomyces toxytricini* (32, 33). In 2019, the Liu group reported the biochemical and functional characterizations of LstAB (34). LstA and LstB are adjacent to each other in the *lst* cluster and are both β-ketoacyl-acyl carrier protein synthase-III (KAS-III)-like enzymes sharing 23% overall sequence identity (Fig. 3*A*) (35). Chemical structure of lipstatin is shown in Figure 3*B*. Attempts to obtain soluble forms of LstA and LstB failed, while the coexpression of these two proteins resulted in the soluble complex LstAB, which could be copurified with only one of them bearing a hexahistidine tag. LstAB shows considerable sequence identity to *E. coli* β-ketoacyl-[acyl-carrier-protein] synthase III, which is also acknowledged as FabH. FabH shares 26% sequence identity with LstA and 18% with LstB. Unlike the typical type of condensation reaction catalyzed by FabH (Fig. 3*C*), LstAB catalyzes the condensation reaction between (3*S*,5*Z*,8*Z*)-3-hydroxytetradeca-5,8-dienyl-CoA and octanyl-CoA or its unsaturated analog, (2*E*)-octenyl-CoA, to afford the respective C_22_ α-branched β-ketoacid products, as shown in Figure 3*D*. A sequence-based homology analysis revealed that LstA contains a catalytic triad composed of C128, H271, and N305. LstA^N305A^ failed to form a soluble complex with LstB, and LstA^C128A^B and LstA^H271A^B lost enzymatic activity. It is noteworthy that the LstAB^E60A^ mutant, though still in a soluble form when expressed, also completely lacked catalytic activity, implying that the LstA and LstB subunits of heterodimeric LstAB enzyme both serve catalytic roles in biosynthesis. Because of the lack of structural information about the LstAB heterodimer, we present the complex model predicted by Alphafold 3, as illustrated in Figure 3*E*. In this predicted structure, LstA forms a tight heterodimer with LstB in a highly similar manner to the FabH homodimer (PDB ID: 5BNS), using the same interface. However, experimental evidence has proved that neither LstA nor LstB is able to form a stable homodimer in solution. This could result from some mutations on the interaction interface that specifically allow heterodimer formation and prevent the formation of homodimers. For the homodimeric FabH enzyme, a symmetric interface is formed by several sets of residues, which are not conserved in LstAB. These mutations on the dimerization interface not only prevent LstA and LstB from forming homodimers but also enable the formation of the active LstAB heterodimer. A phylogenetic analysis revealed that LstA is related to KAS-III homologues, including OleA (36), PpyS (37), and FabH (38), while LstB appears to be more independent in its evolution and is less related to the KAS-III family enzymes. Counterparts of LstB are widely present in bacterial genomes and always coclustered with genes encoding LstA. This cassette might have evolved as an enzyme cascade for assembling long aliphatic chains to provide backbones for the generation of various lipstatin-like natural compounds.

## Hinokiresinol synthase HRSαβ

**Figure 4:**
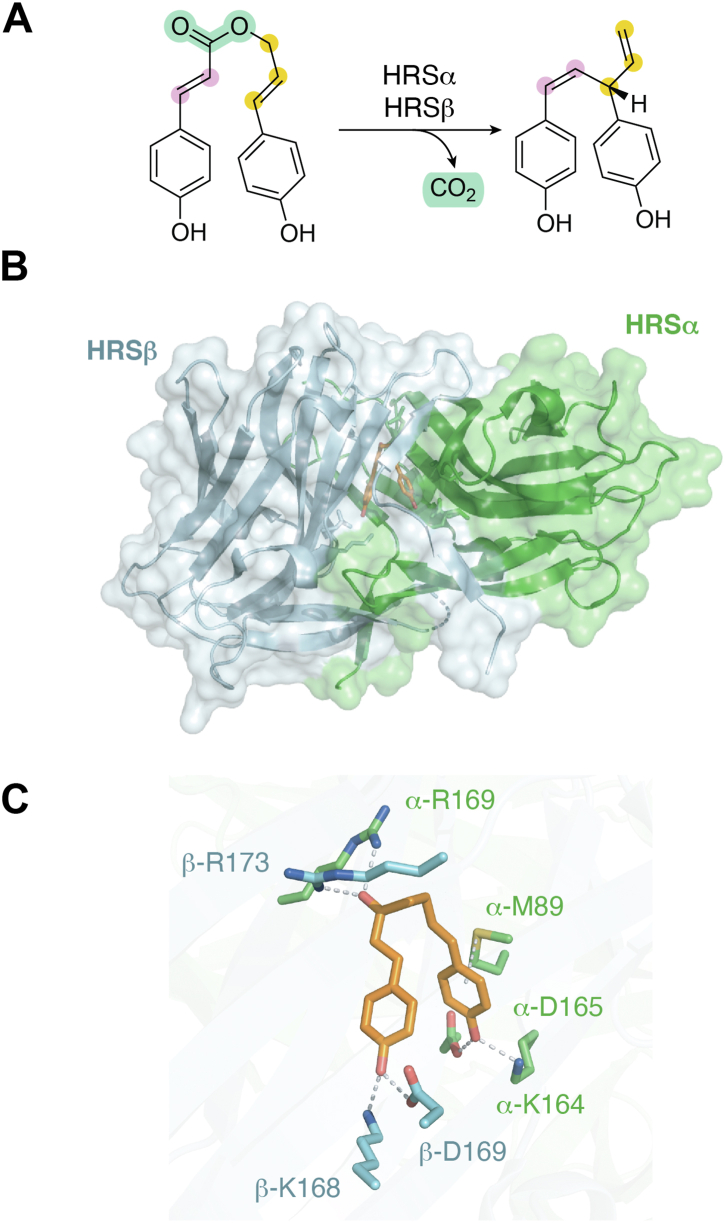
**Heterodimeric hinokiresinol synthase HRSαβ.***A*, conversion reaction of 4-coumaryl 4-coumarate into (*Z*)-hinokiresinol by HRSαβ. *B*, cocrystal structure of HRSαβ with a substrate analog (PDB ID: 8JMR). *C*, binding of the substrate analog by HRSαβ. *Dashed lines* indicate hydrogen bonding interactions between residues of HRSαβ and the bound inhibitor. HRS, hinokiresinol synthase; PDB, Protein Data Bank.

Hinokiresinols are norlignans, produced by plants including *Asparagus officinalis* and *Cryptomeria japonica*, with antifungal activity and serve as chemical defense agents in response to stress. In 2007, the Umezawa group reported the discovery of the hinokiresinol synthase (HRS) from *A. officinalis* as a complex consisting of two subunits, HRSα and HRSβ, which share 54% sequence identity (39). As the heterodimer, HRSαβ catalyzes the conversion of 4-coumaryl 4-coumarate into (*Z*)-hinokiresinol (Fig. 4*A*). In 2023, the Abe group reported structural and catalytic insights of HRSαβ^,^ (40). When coexpressed in *E. coli*, authentic HRSα could be copurified with N-His_6_-tagged HRSβ. This protein mixture also coeluted in a size-exclusion chromatography analysis. The X-ray crystal structure of HRSαβ revealed a tightly bound heterodimer with buried surface area of 2337 Å^2^ (PDB ID: 8JMQ). In the cocrystal structure of HRSαβ and a synthesized analogue of the substrate, 4-coumaryl 4-coumarate (PDB ID: 8JMR), shown in Figure 4*B*, an exquisite substrate binding pocket is sandwiched between the HRSα and HRSβ subunits. The M89, K164, D165, and R169 residues of HRSα, together with the R173, K168, and D169 residues of HRSβ and some hydrophobic residues from each subunit, form the substrate-binding site (Fig. 4*C*), with their significance verified by site-directed mutagenesis experiments. Although the investigations yielded catalytic insights, the evolutionary advantage of the heterodimer remained unidentified. It is possible that these genes are transcribed and regulated separately to provide more precise protective responses for plants, but this conjecture requires experimental evidence to verify its validity.

## 2-Aminoisobutyric acid hydroxylase AibH1H2

**Figure 5:**
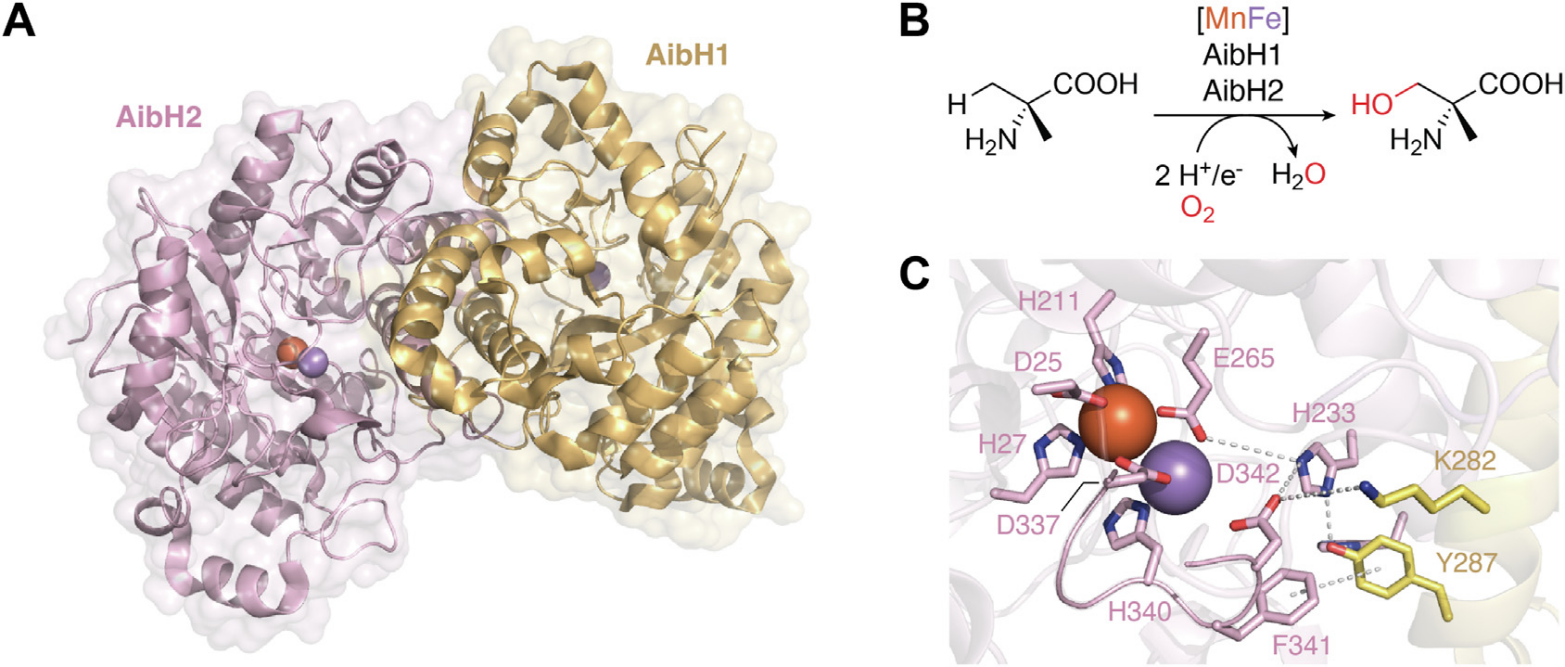
**Heterodimeric 2-aminoisobutyric acid hydroxylase AibH1H2.***A*, complex structure of AibH1H2 (PDB ID: 8FUN). Metal ions are shown as *colored spheres* (Fe^2+^ is *orange* and Mn^2+^ is *purple*). Buried surface area of the interface is 2792 Å^2^. *B*, oxidation of α-aminoisobutyric acid into α-methyl-(D)-serine catalyzed by AibH1H2. *C*, metal binding center for the [MnFe] cluster in AibH1H2. *Dashed lines* indicate hydrogen bonding interactions between residues of AibH1H2. PDB, Protein Data Bank.

In 2021, the Ogawa group found that, in the initial step of α-aminoisobutyric acid (Aib) conversion in *Rhodococcus wratislaviensis* C31 to 06, a three-component monooxygenase system consisting of AibF, AibG, and AibH1H2 undertakes the role of selective terminal hydroxylation of Aib into α-methyl-(D)-serine (Fig. 5*B*), in which AibH1H2 serves as the hydroxylase and AibG and AibF function as a ferredoxin and a ferredoxin:NAD^+^ oxidoreductase, respectively (41). In 2023, subsequent research by Powell et al., showed that AibH1H2 exhibits strict metal dependency on manganese and utilizes an unusual heterometallic Mn/Fe cofactor to perform its catalytic activity in the heterodimeric form (Fig. 5*A*) (42). The AibH2 subunit is a structural homolog of a hydroxylase, PtmU3 (43), which was repurposed from the amidohydrolase superfamily involved in platensimycin biosynthesis. Utilizing a specific [FeMn] cluster, AibH2 mediates the hydroxylation of Aib, while AibH1 more likely serves to maintain the structure of the catalytic complex, since mutations of its metal chelating residues result in an insoluble AibH1H2 complex. Although a metal binding center is observed in AibH1, experimental evidence suggested that this metal only functions as a structural element for maintaining the quaternary structure of the correct protein fold (41, 42). Bioinformatics searches revealed that AibH2 belongs to the PF4909 family and its homologs do not always coexist with AibH1-like proteins, implying that AibH1 might only play a specific auxiliary role in the heterodimeric AibH1H2 complex. Although the auxiliary role of AibH1 remains elusive, we can make some assumptions based on the cocrystal structure. As shown in Figure 5*C*, in the active center of AibH2, the [MnFe] cluster is chelated by the D25, H27, H211, E265, D337, H340, and D342 residues of AibH2. Among these AibH2 residues, D337, H340, and D342 are located on a flexible loop that is close to the dimerization interface with AibH1. The AibH2^T336-F343^ loop interacts with AibH1 by forming hydrogen bonds between AibH2^D342^ and AibH1^K282^, with a parallel-displaced π–π stacking between AibH2^F341^ and AibH1^Y287^ further enhancing the interaction. The interaction with AibH1 might cause certain conformational changes in AibH2, especially in the flexible loop region, to form the metal binding center as well as the substrate binding pocket. The discovery and characterization of heterodimeric AibH1H2 has enriched our understanding of oxygenases with complex metal centers.

## C-glycoside deglycosidases

**Figure 6:**
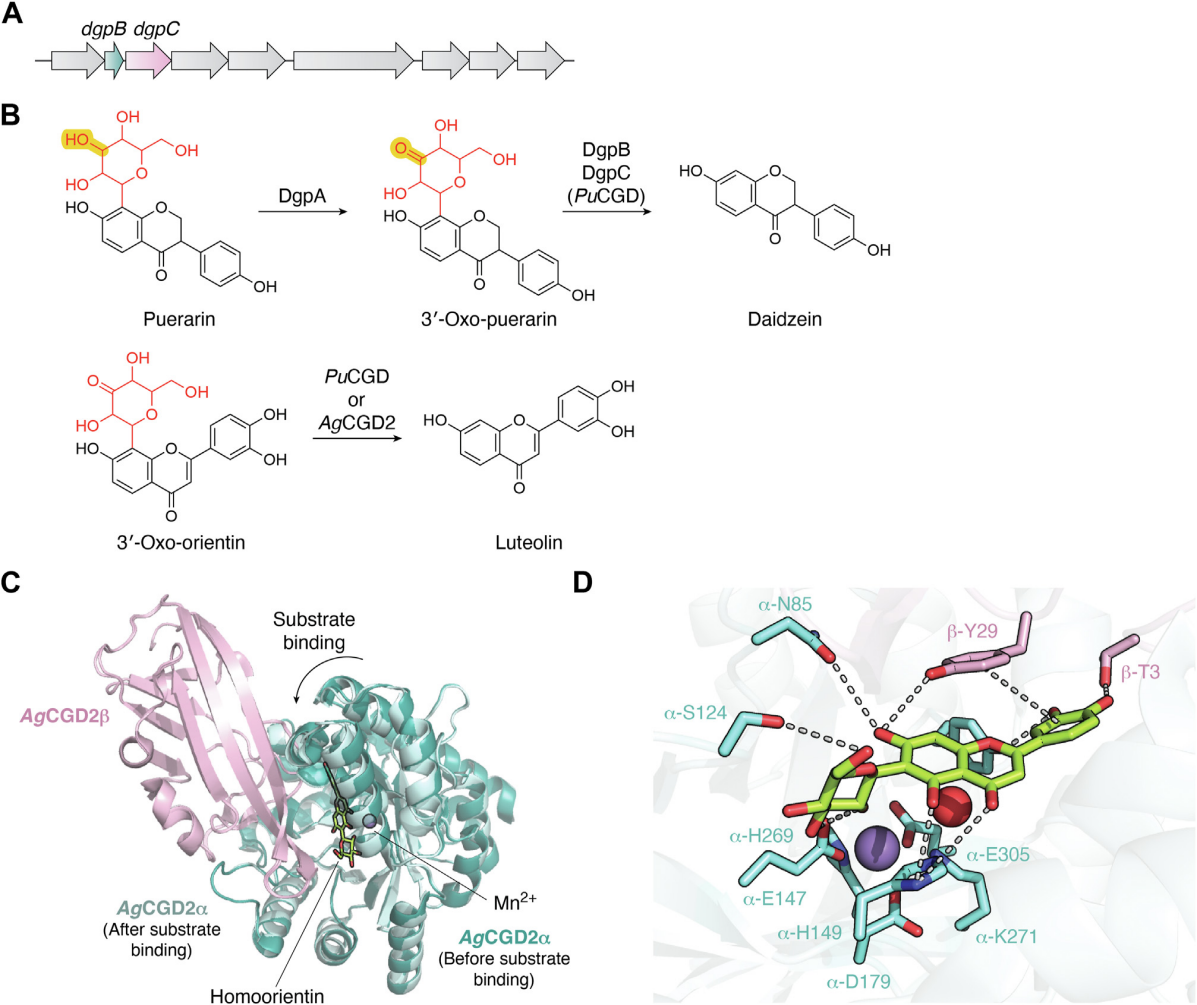
**Heterodimeric C-glycoside deglycosidase.***A*, the *dgp* cluster responsible for C-deglycosylation of flavonoid C-glycosides, from a PUE bacterial strain. *B*, two-step C-C bond cleavage reaction by the oxidoreductase DgpA and the C-deglycosidases DgpBC (*Pu*CGD), and the deglycosylation reaction catalyzed by *Ag*CGD2. *C*, superimposition of apo crystal structure of *Ag*CGD2 (PDB ID: 7DNM) and cocrystal structure of *Ag*CGD2 bound with homoorientin (PDB ID: 7DNN). Buried surface area of dimerization interface is 1521 Å^2^. *D*, binding of Mn^2+^ and homoorientin in the active site of *Ag*CGD2 (PDB ID: 7DNN). *Dashed lines* indicate hydrogen bonding interactions between residues of *Ag*CGD2 and Mn^2+^ or homoorientin. PDB, Protein Data Bank.

Deglycosylation reactions by intestinal microbes play an important role in the absorption and activation of various bioactive compounds, such as drug glucuronide metabolites and plant-derived natural products (44, 45, 46). The *dgp* cluster (Fig. 6*A*), which was identified from a PUE bacterial strain, is responsible for the C-deglycosylation of flavonoid C-glycosides (47, 48). As shown in Figure 6*B*, after the oxidoreductase DgpA oxidizes the glucoside moiety of puerarin, the heteromeric complex DgpBC (also referred to as *Pu*CGD, puerarin C-glycoside deglycosidase) cleaves the sugar moiety to generate daidzein. In 2021, the Kobayashi group and the Abe group reported a detailed structure-based mechanistic analysis of the C-glycoside deglycosidases (49). DgpBC was confirmed to exist as an α4β4 heterocomplex in solution, by copurification and a size-exclusion chromatography with multi-angle light scattering analysis. While DgpC is a sugar isomerase/epimerase-like enzyme, DgpB remains poorly annotated according to a BLAST (Basic Local Alignment Search Tool) analysis. A close homolog of *Pu*CGD from *Microbacterium trichothecenolyticum* was identified and designated as *Ag*CGD2, and deglycosylates 3′-oxo-orientin to generate luteolin. The crystal structures of *Ag*CGD2 (PDB ID: 7DNM) and its cocrystal (PDB ID: 7DNN) with a substrate analog, homoorientin, were determined as shown in Figure 6, *C* and *D*. The α subunit, which is homologous to DgpC, binds the catalytically essential Mn^2+^ ion together with the substrate *via* several residues close to the αβ interface, and the β subunit, which is homologous to DgpB, also binds the substrate by hydrogen bonding and π–π stacking. Interestingly, the lid domain of the β subunit moves by ∼3 Å and rotates by ∼18 degrees toward the active site cavity of the α subunit upon substrate binding (Fig. 6*C*). The formation of the C-glycoside deglycosidase heterocomplexes facilitates substrate recognition and catalysis.

## Phosphonopyruvate decarboxylase RhiEF

**Figure 7:**
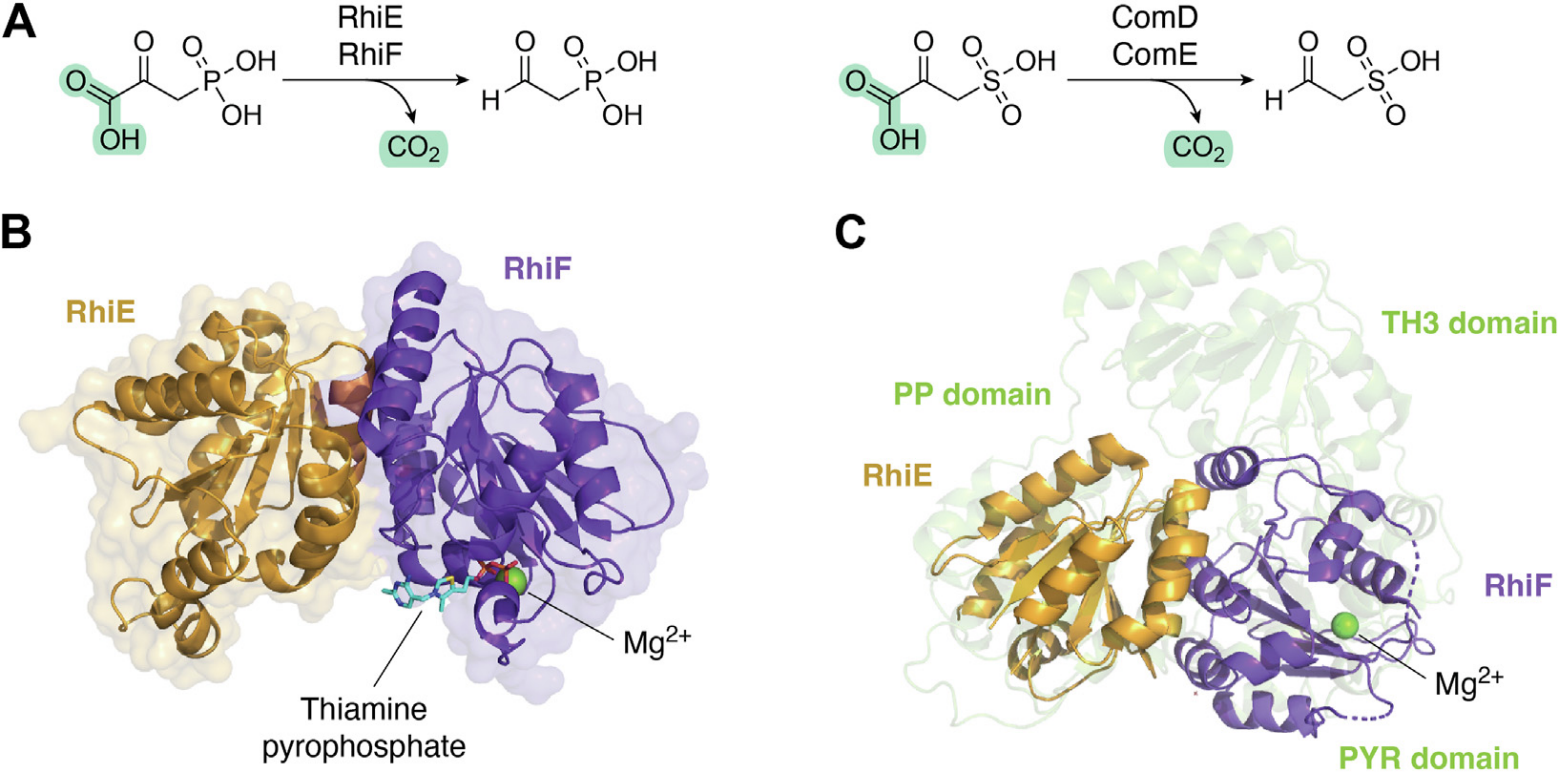
**Heterodimeric phosphonopyruvate decarboxylase RhiEF.***A*, decarboxylation reactions catalyzed by the phosphonopyruvate decarboxylase RhiEF and ComDE toward their respective substrates. *B*, complex structure of heterodimeric RhiEF and its cofactor thiamine pyrophosphate (PDB ID: 9IZ4). *C*, overlay of the overall apo crystal structure of RhiEF (PDB ID: 9IZ3). with yeast pyruvate decarboxylase (PDB ID: 1QPB). PDB, Protein Data Bank.

In 2000, two adjacent genes, *comD* and *comE*, in the *Methanococcus jannaschii* chromosome were identified (50) as encoding enzyme products with decarboxylation activity in the coenzyme M biosynthetic pathway. Recently, the heterodimeric phosphonopyruvate decarboxylase RhiEF was identified in 2024 by Nakamura *et al.* (51), and its subunits, RhiE and RhiF are homologous to ComD and ComE, with 47% and 52% sequence similarities, respectively. These enzymes collaboratively function in the rhizocticin biosynthetic pathway (52), converting phosphonopyruvate (PnPy) into phosphonoacetaldehyde (PnAA), as shown in Figure 7*A*. These enzymes were copurified and their cocrystal structure was solved by X-ray diffraction (PDB ID: 9IZ3, Fig. 7*B*). Notably, the buried surface area of the RhiEF complex overtakes 12% (2000 Å^2^) of the monomer surface area. Although most PnPy decarboxylases only consist of a single polypeptide chain (53), *rhiE* and *rhiF* are separated as two open reading frames in the *rhi* cluster, resembling the N-terminal and C-terminal portions of single chain PnPy decarboxylases. RhiEF is thiamin pyrophosphate (TPP) dependent and binds this cofactor using its heterotetrameric interface. RhiE and RhiF shares 34% and 36% sequence similarity to the pyrophosphate-binding (PP) domain and the PYR domain of yeast pyruvate decarboxylase (PDB ID:1QPB). A structural alignment analysis (Fig. 7*C*) indicated that RhiE and RhiF resemble the PYR (pyrimidine-binding) and PP (pyrophosphate-binding) domains, respectively, of TPP-dependent enzymes, such as the mentioned yeast pyruvate decarboxylase. Despite the similarities in the structures and enzymatic activities, the biological meaning of the separated reading frames of the *comD*/*comE* and *rhiE*/*rhiF* genes remains enigmatic. However, an evolutionary analysis revealed that ComD/ComE is a pair of ancestral TPP-dependent enzymes generated from a common ancestor protein and arose from gene duplication (54, 55). These two subunits were subsequently fused into a single polypeptide chain and then other small subunits, such as the TH3-domain, for allosteric regulation were joined together by later evolutionary events. The fusion of the two PnPy decarboxylase domains might improve the stability and increase the enzyme efficiency over the separately expressed proteins, but more experimental evidence is required to better understand the biochemical rational underlying the fusion of a heterodimeric enzyme into a single polypeptide enzyme.

## Lexapeptide dehydratase LxmKY

**Figure 8:**
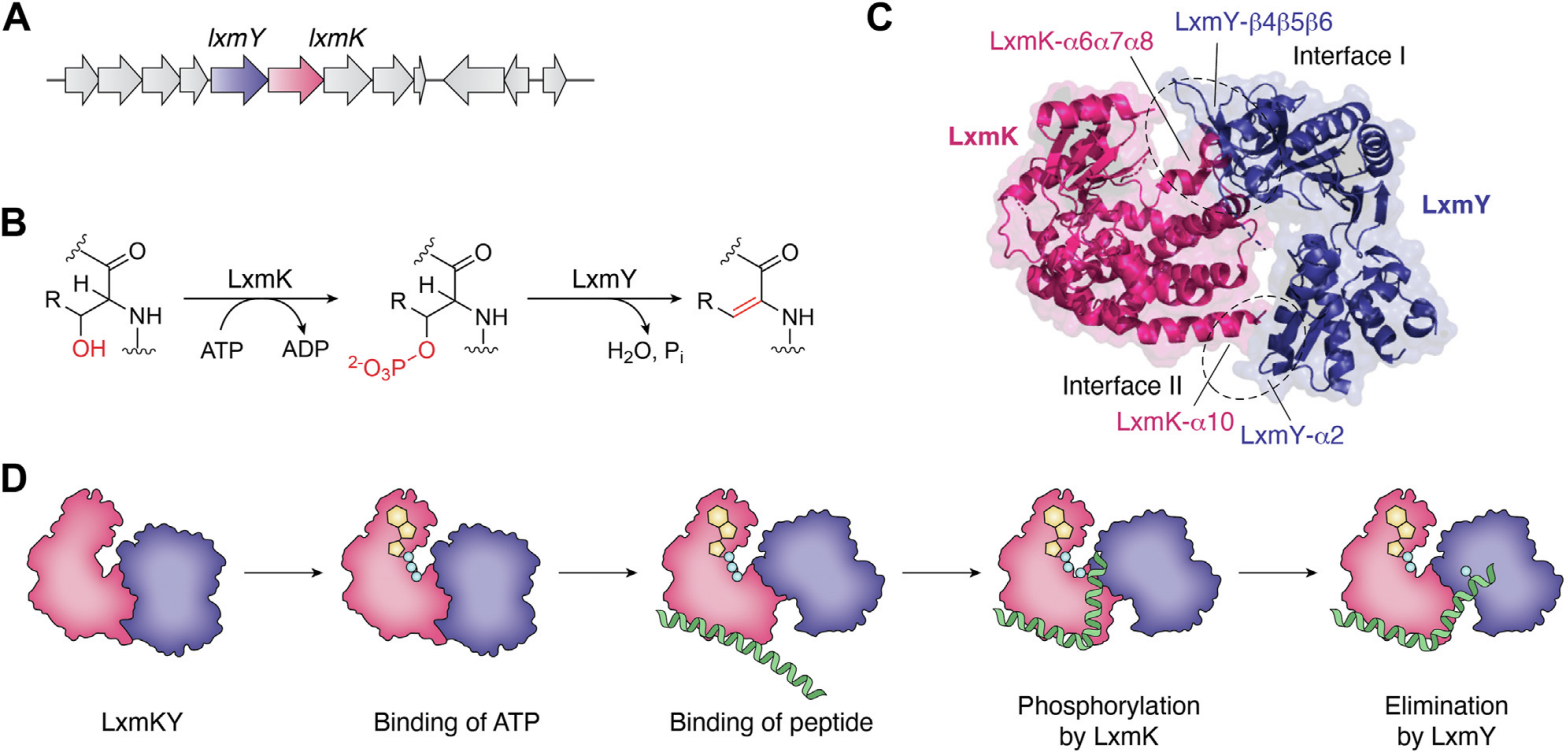
**Heterodimeric lexapeptide dehydratase LxmKY.***A*, the *lxmY* and *lxmK* genes in the lexapeptide-encoding biosynthetic gene cluster. *B*, stepwise conversion in the Dha/Dhb installation reaction by LxmKY. *C*, complex structure of the LxmKY heterodimer (PDB ID: 9DK1) and the two interfaces. *D*, schematic representation of proposed mechanism of Dha/Dhb installation by LxmKY (56). PDB, Protein Data Bank.

Lexapeptide, a lanthipeptide produced by *Streptomyces rochei* exhibiting antibiotic potency against *Staphylococcus aureus*, is synthesized by a cluster consisting of seven genes, as shown in Figure 8*A* (56). Before the maturation of the precursor peptide LxmA, eight 2,3-dehydroalanine (Dha) or (*Z*)-2,3-didehydrobutyrine (Dhb) residues must be installed. This process is mediated by the LxmK/LxmY heterodimer, with kinase and lyase functions, respectively, to dehydrate serine or threonine residues into Dha or Dhb (Fig. 8*B*). The Bashiri group reported mechanistic insights of LxmKY in 2024 (56). When these enzymes were separately expressed, LxmK could only remain soluble after fusion with a maltose binding protein tag, and LxmY formed aggregates that eluted in the void volume during gel filtration purification. The coexpression of LxmK and LxmY not only enhanced the stability but also increased the yield for both of these enzymes. The formation of a stable LxmKY heterodimer was verified by a size-exclusion chromatography analysis as well as a multiangle laser light scattering analysis. LxmK is a homolog of aminoglycoside phosphotransferase, which is characterized by a conserved EPKLK fold. For LxmY, apart from its homology with HopA1 effector proteins, which are secreted by pathogenic bacteria to dephosphorylate certain regulatory proteins in the host in order to hamper the immune response, a small domain (LxmY^104-160^) was identified as a nonconserved protein. As shown in Figure 8*C*, two interfaces in between the subunits were observed in the cocrystal structure of LxmKY (PDB ID: 9DK1) with a total buried surface area of 873 Å^2^. The α6−α8 helices of LxmK and the twisted antiparallel β-sheet domain in LxmY contributed to most of the interactions, forming the major interface with participation from the above mentioned LxmY^104-160^ domain. A hydrophobic interface is formed between LxmK^L174^, LxmK^W176^, LxmK ^V179^ and LxmK ^M184^ and LxmY^V136^, LxmY^V132^, and LxmY^L165^, while a salt bridge between LxmY^R55^ and LxmK^E293^ connects the other end of each peptide chain. These two interaction interfaces create a cavity between LxmK and LxmY for binding the precursor peptide substrate, LxmA. The Alphafold 3-predicted complex structure of LxmKY and LxmA revealed that the leader peptide part of LxmA binds to the C-terminal lobe of LxmK by hydrophobic interactions. However, sampling of the complex composed of the core peptide part of LxmA with LxmKY resulted in various conformations, in which LxmA core peptide bound to the LxmK kinase active site or the LxmY lyase active site, implying the dynamic conformation of the substrate-enzyme complex. Soaking LxmKY crystals with ATP and MgCl_2_ resulted in the binding of ATP and 2 Mg^2+^ ions in the LxmK active site, which ordered the previously disordered LxmK^F42-A49^ G-loop in the apo crystal, sealing the ATP binding site. Once the LxmA core peptide part is phosphorylated by LxmK, it moves to the LxmY active site for the subsequent elimination reaction, as proposed in Figure 8*D*. The hydrophobic residues LxmY^F169^ and LxmY^Y171^ guard the active site of LxmY to protect the elimination reaction from water. Allosteric regulation of the LxmK loops directs the reaction to occur in a precise, stepwise manner. The formation of the LxmKY heterodimer enables LxmA to be dehydrated without leaving the cavity of the complex, which enhances the efficiency and velocity of the overall reaction.

## Perspective

The rapid development of biochemical and structural biology techniques, such as size-exclusion chromatography with multi-angle light scattering, isothermal titration calorimetry, and cryo-EM, has enabled an increasing number of heteromeric enzymes to be purified, characterized, and understood based on structurally supported mechanisms. The formation of these complexes plays a crucial role in biocatalytic processes, by assisting in the stabilization of the overall structure, recognizing substrates, adjusting conformations with allosteric regulations, and efficiently organizing multistep reactions.

In this review, we present several representative heteromeric enzymes discovered in recent years. These heteromers are formed for a variety of reasons and with different insights. In some cases, only one subunit in the heterodimer undertakes the role of catalysis, while another subunit functions as an auxiliary protein, as in the TlxIJ, AibH1H2, and MNIO enzymes mentioned above. Although TlxI and AibH1 are proposed to contribute to the enzymatic reaction by maintaining a preferred conformation for their catalytic partners, the noncatalytic proteins in MNIOs more likely serve as guides for the precursor peptide substrates. In the cases of HRSαβ and LstAB, both subunits are enzymatically involved and catalyze the conversions together. For C-glycoside deglycosidases, the β subunit contributes to substrate recognition and binding, while for RhiEF and LxmKY, the two subunits bind with substrates in a stepwise manner to facilitate catalysis.

The evolutionary behavior underlying the transition between heterodimeric enzymes and naturally occurring fusion chimeras is also interesting and noteworthy (34, 51). Although certain advantages of separately expressed enzymes and fusion enzymes could be mentioned (57), it is important to learn more information from phylogenetic and bioinformatic analyses to understand the order and time scale of the evolution of these enzymes. Fusion or separation is the crucial decision for the survival of different forms of life (58).

Natural wisdom is valuable for protein design and engineering. In addition, understanding the emergence of heterodimeric enzymes on the evolutionary scale is also very helpful to clarify the developmental process of some natural chimeric proteins. When analyzing gene clusters encoding certain secondary metabolites, the possible existence of heterodimeric proteins should be considered, and this can be greatly facilitated by rapidly developing sequence-based protein complex prediction technologies, such as Alphafold 3 (23). Explorations of heterodimeric enzymes will provide deeper understanding of biosynthetic reactions, and foster the development and application of more efficient and precise enzyme tools.

## Conflict of interest

The authors declare that they have no conflicts of interest with the contents of this article.
